# The reporting of disproportionality analysis in pharmacovigilance: spotlight on the READUS-PV guideline

**DOI:** 10.3389/fphar.2024.1488725

**Published:** 2024-11-27

**Authors:** Michele Fusaroli, Francesco Salvo, Charles Khouri, Emanuel Raschi

**Affiliations:** ^1^ Department of Medical and Surgical Sciences, Alma Mater Studiorum - University of Bologna, Bologna, Italy; ^2^ Université de Bordeaux, INSERM, BPH, Team AHeaD, U1219, Bordeaux, France; ^3^ CHU de Bordeaux, Service de Pharmacologie Médicale, INSERM, U1219, Bordeaux, France; ^4^ University Grenoble Alpes, Pharmacovigilance Unit, Grenoble Alpes University Hospital, Grenoble, France; ^5^ University Grenoble Alpes, Inserm U1300, HP2, Grenoble, France

**Keywords:** disproportionality analysis, individual case safety reports, pharmacovigilance, adverse drug reactions, signal detection

## Abstract

Disproportionality analyses are the most-commonly used study design used in the post-marketing phase to detect suspected adverse drug reactions in individual case safety reports. Recent years have witnessed an exponential increase in published articles on disproportionality analyses, thanks to publicly accessible databases. Unfortunately, this trend was accompanied by concerns on lack of transparency and misinterpretation of results, both generating unjustified alarm and diluting true signals into overwhelming noise. The READUS-PV guideline for reporting disproportionality analysis was developed to tackle this emerging issue. In this perspective article, we describe the rationale behind the development of the READUS-PV guideline, the first collaborative initiative to harmonize the reporting of disproportionality analyses. The adoption of the checklists will assist researchers, regulators, and reviewers in the reporting, assessment, and publication of disproportionality analyses. Acknowledging the challenges ahead of effective implementation, we advocate for a global endorsement by Pharmacology Journals. A wide dissemination of the READUS-PV guideline is crucial to foster transparency and reproducibility of pharmacovigilance research, supporting an effective exploitation of disproportionality analysis among other irreplaceable post-marketing research tools to ensure drug safety.

## 1 Disproportionality analysis: Basic concepts

Individual case safety reports (ICSRs) of suspected adverse drug reactions (ADRs) are the main post-marketing data source used for detecting new safety signals, possibly resulting in regulatory measures such as safety-related labeling changes. ICSRs, collected in dedicated archives, are either spontaneously reported by healthcare professionals and patients or generated within active surveillance activities (Tau et al., 2019; Croteau et al., 2022; Sartori et al., 2023).

**FIGURE 1:**
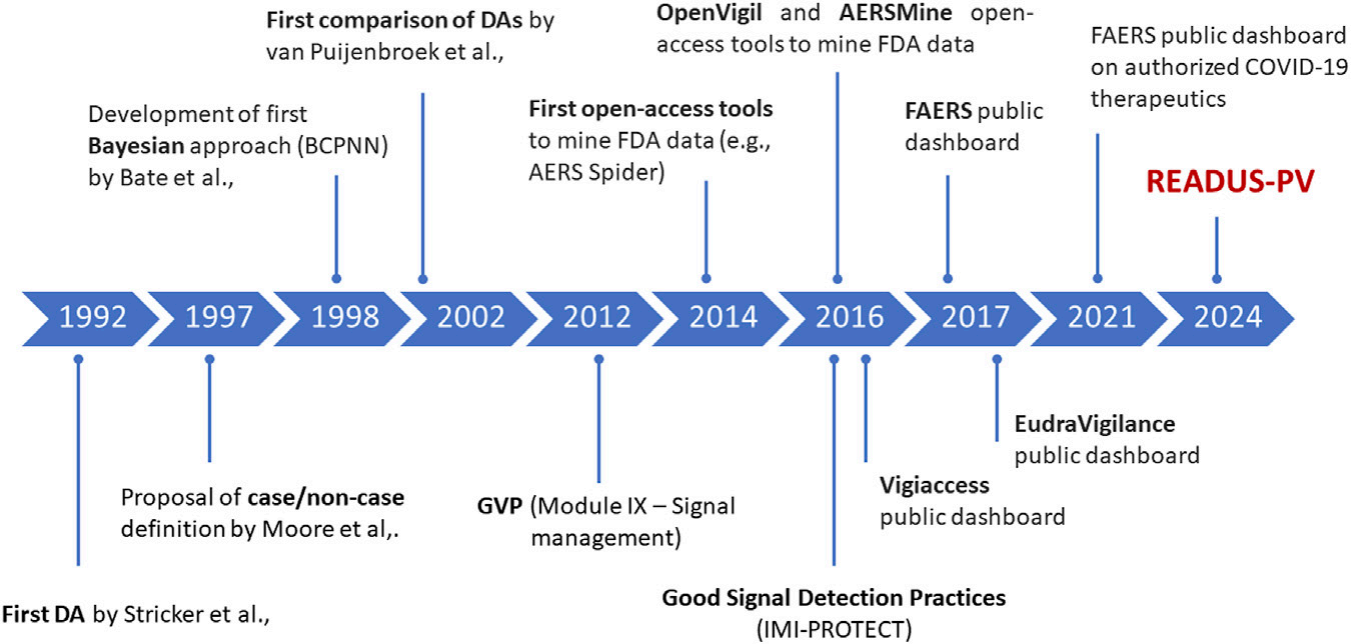
Milestones in the development of disproportionality analysis and access to major pharmacovigilance databases. BCPNN, Bayesian Confidence Propagation Neural Network; DA:, disproportionality analysis; FAERS, FDA Adverse Event Reporting System; FDA, Food and Drug Administration; GVP, Good PharmacoVigilance Practices [Regulation (EU) No 1027/2012 and Directive 2012/26/EU].

Disproportionality analysis (DA) is a study design based on ICSRs and developed to identify drug-event combinations for which the count of observed cases exceeds the count of expected cases (generating the so-called signal of disproportionate reporting–SDR). The first published attempt to evaluate the extent of reporting using ICSRs was by Bruno Stricker in 1992, who explored the association between cefaclor exposure and serum sickness reactions (Stricker and Tijssen, 1992). Five years later, Moore et al., (Moore et al., 1997), introduced the concept of “case/non case method” when performing DA, to distinguish it from the case-control design used in pharmaco-epidemiological research (Figure 1).

Several DA approaches exist today, distinct by underlying assumption, rationale, and calculation. Despite efforts to identify general recommendations on the best approach, no absolute gold standard currently exists (Bate et al., 1998; van Puijenbroek et al., 2002; Wisniewski et al., 2016; Ding et al., 2020; Fusaroli et al., 2024c). Instead, the choice of the approach should explicitly depend on various factors, including the research purpose, database size, drug characteristics and ADR features (Jiao et al., 2024).

Although these approaches are known as “quantitative” methods (Bate and Evans, 2009), two major issues need to be considered. First, due to the lack of information about reporting rates and drug exposure, the incidence of an ADR (i.e., measures of risk) cannot be estimated. Second, because of numerous biases affecting observational data, the presence of an SDR *per se* does not necessarily imply a causal drug-event relationship, nor does the absence of a statistically significant result necessarily disprove a possible ADR [see (Cutroneo et al., 2024; Fusaroli et al., 2024b) for a thorough discussion about bias in DA and possible approaches to partly address them].

DAs cannot be used as a standalone approach to provide clinical recommendations such as direct safety comparisons between drugs (Michel et al., 2017; Raschi et al., 2018). The SDRs generated by a DA should be complemented by a careful case-by-case analysis as the first step within the signal management process, and require further thorough evaluation through comprehensive assessments involving an in-depth examination of biological plausibility, safety data from other drugs in the same class, information from available clinical trials and pharmacoepidemiologic studies, preclinical data, and the application of causality frameworks to substantiate and clarify any causal relationships (Hammad et al., 2023). The hypotheses generated by a DA frequently requires evaluation through analytical pharmaco-epidemiological approaches, including case-control or cohort studies, which do allow risk assessment (Cutroneo et al., 2024).

## 2 Disproportionality analysis: A “trendy” study design in the recent literature

In recent years, we have witnessed an exponential increase in the number of publications on DAs (Wang et al., 2021), with articles growing at an almost exponential rate from the year 2017 onwards (Loke et al., 2024). These published DAs came mostly from academia (Sartori et al., 2023), with a considerable interest from “clinical Journals”, notably in the field of oncology (Raschi et al., 2020; Sa et al., 2023).

Multiple reasons can be identified behind this publication trend, including: 1) the virtuous initiatives of regulators to allow public access to ICSRs databases through both raw data and online interfaces (e.g., the Food and Drug Administration Adverse Event Reporting System public dashboard and Vigiaccess); 2) the widespread availability of online user-friendly tools for quick database queries, though often lacking transparency in preprocessing and limiting in-depth custom analyses (Giunchi et al., 2023); 3) the ease of data access and the lack of formal ethics approval requirements due to data anonymization; 4) the lack (up to now) of rigorous reporting practices specifically tailored to DA.

On one hand, this surge in the number of published DAs denotes an increased awareness on the importance of post-marketing surveillance for drug safety; on the other hand, this trend poses challenges and significant burden to various stakeholders, including pharmaceutical companies (scanning the literature as part of pharmacovigilance requirements), regulators and policymakers (possibly using results from DAs to drive regulatory measures), researchers (deciding whether an SDR needs evaluation through additional observational research), and clinicians (potentially adopting DA-driven clinical decisions in their everyday practice).

## 3 Background to the READUS-PV guideline: a critical literature appraisal

Over the years, a general skepticism has emerged on the role and usefulness of DAs. Due to the unique nature of ICSRs and their traditional use being limited to hypothesis generation, there has been little academic focus on refining the study design of DAs. In 2011, the *British Journal of Clinical Pharmacology* raised the debate on the benefits of publishing results of DAs in medical journals (de Boer, 2011; Montastruc et al., 2011). Later, the terms “apophenia” and “pharmacovigilance syndrome” have been coined, namely, the incorrect perception and interpretation of statistically significant disproportionality to infer causal association (Gagne, 2014; Greenblatt, 2015). All these aspects might explain, at least partially, why the vast majority of SDRs remains unnoticed by researchers and are not corroborated by subsequent published research (Dhodapkar et al., 2022; Loke et al., 2024). At the same time, they might generate unjustified alarms among clinicians, as exemplified by the case of pancreatitis with GLP-1 receptor agonists (Moore, 2011). In the following paragraphs, we will discuss three main challenges when performing DAs: implementation, documentation, and interpretation.

Performing an exhaustive DA study requires not only calculating an association, but rather implementing a series of steps to provide a first assessment of the SDR and enhance its validity. Implementing these steps–defining the hypothesis within existing knowledge, designing a methodologically robust study with justified strategies (possibly integrating case-by-case and complementary analyses), and interpreting results in context–can involve extremely heterogenous approaches, potentially leading to various outcomes (Fusaroli et al., 2023). Although the IMI PROTECT project provided a set of 39 pragmatic recommendations that can be used as a guideline for implementing signal detection using DAs (Wisniewski et al., 2016), there are still a number of challenges and unsettled research issues towards proper integration of DA into the wider pharmacovigilance landscape (Fusaroli et al., 2024c). For example, the selection of the most suitable comparator remains a significant challenge (Gravel and Douros, 2023; Gravel et al., 2024), even if efforts have been made to develop a system that helps predict and address biases in DAs under certain assumptions (Fusaroli et al., 2024b). Other considerations concern the lack of an *a priori* power calculation for routine DAs employed in signal detection, the absence of pre-specification of analyses in protocols (hence the likely production of overlapping research), and the inflation of the risk of false-positive findings from multiple statistical comparisons (Gaucher et al., 2023; Naudet et al., 2024).

Documenting a DA (namely, full transparency on how the study was performed and justification of methodological choices) is challenging, in the absence of a guideline. Recently, a meta-research study questioned the reproducibility of DAs, with over 75% of published DAs failing to report essential elements needed to understand and replicate the analyses and results, including the thresholds for SDR definition and the comparator (Khouri et al., 2021b).

Finally, interpreting DAs is also a challenge. A meta-research study estimated that over 66% of published DAs over-interpreted their findings, the so-called “spin”, in the form of: a) claiming for causality without addressing potential biases, b) calculating incidence and risk assessment based on ICSRs alone, and c) providing unjustified clinical recommendations (Mouffak et al., 2021; Khouri et al., 2023). A potential explanation for this misinterpretation lies in the fact that, although case/non-case approaches resemble case-control designs, they differ in the type of data they use and the biases that affect them. Some meta-research studies found a “significant correlation” between relative risks from pharmacoepidemiologic studies or meta-analyses of clinical trials and estimates from DAs, advocating for DAs as a possible early indicator of an ADR’s potential clinical importance (Maciá-Martínez et al., 2016; Beau-Lejdstrom et al., 2019). However, these correlations tend to be inflated, particularly when biases are not addressed, and vary greatly depending on the drug and ADR type (Khouri et al., 2021a). Therefore, it is strongly advised not to use disproportionality measures *per se* as a risk measure (Khouri et al., 2023), nor to combine them with other data in safety meta-analysis without further considerations (Raschi et al., 2016; Fusaroli et al., 2024a).

In this intricate scenario, in which implementation, documentation and interpretation of DAs are often of poor quality, the credibility of DAs has been progressively undermined, and we recently called for a concerted effort to harmonize the conception, design and reporting of DAs (Raschi et al., 2022).

## 4 Creation, development, and overview of the READUS-PV guideline

The REporting of A Disproportionality analysis for drUg Safety signal detection using individual case safety reports in PharmacoVigilance [READUS-PV] guideline is the first aid for reporting the results of DAs in articles and abstracts (Fusaroli et al., 2024d; Fusaroli et al., 2024e). As a matter of fact, ICSRs have unique features that cannot be adequately captured by existing reporting guidelines for observational and pharmaco-epidemiological studies such as STROBE and RECORD-PE. Only preliminary proposals were previously provided towards minimum reporting requirements, although in the form of discussion and mainly focused on specific ADRs such as liver injury (Raschi et al., 2018; 2023). Importantly, READUS-PV guideline does not provide recommendations to conduct DAs and should be viewed in conjunction with the previous IMI PROTECT project (Wisniewski et al., 2016), and other methodological considerations by Regulators (European Medicines Agency, 2017), with the unsettled issues discussed in the previous section.

The READUS-PV guideline is the result of a worldwide interprofessional collaboration. Through an open-text survey followed by a modified Delphi process, 34 pharmacovigilance experts from academia, drug companies and regulatory agencies reached a consensus on a checklist of 14 items (plus four additional items for the abstract) recommended for consideration when reporting a DA. A glossary of terms is also consistently provided throughout the READUS-PV guideline to avoid misunderstanding, in particular, the distinction between SDR [a statistical association between medicinal product(s) and event(s) identified by any disproportionality analysis within an ICSR database] and safety signal [information that arises from one or multiple sources, including observations and experiments, which suggests a new potentially causal association or a new aspect of a known association between medicinal product(s) and adverse event(s)]. The Panel agreed on the idea that a mere DA–e.g., not contextualized within the existing knowledge and not integrated with a case-by-case analysis–is of poor value to the scientific and regulatory community.

The READUS-PV checklists can be downloaded in the relevant website (https://readus-statement.org/) and in the Equator network website (https://www.equator-network.org/reporting-guidelines/the-reporting-of-a-disproportionality-analysis-for-drug-safety-signal-detection-using-individual-case-safety-reports-in-pharmacovigilance-readus-pv-development-and-statement/), and can potentially be compiled and submitted together with the manuscript to simplify the retrieval of information (Fusaroli et al., 2024d). An extensive explanation of how the items could be reported, together with essential elements and examples of good reporting, is provided in a companion article (Fusaroli et al., 2024e). These examples have been created using ChatGPT 3.5, using the description of the item as a prompt, with appropriate changes to comply with the READUS-PV guideline and based on authors’ experience.

Briefly, the 14 items comprehensively cover all essential reporting aspects. The *title* should clearly identify the type of data and archive. The *background* and aim should make explicit the rationale underlying the implementation of a DA, including the explanation of how a DA can fill the knowledge gap as compared to existing evidence. The *methods* should define the study population, justify all the operative choices for data pre-processing and analysis [e.g., the definition of “cases”, the selection of “non cases” (comparator), grounding any sensitivity analysis on expected biases], and specify whether and how a case-by-case evaluation was performed. All *results* should be presented, also considering a flow diagram, supporting an independent interpretation by the reader. In the *discussion*, after presenting a summary of key results contextualized within the existing literature, external validity and relevance for clinical practice and research implications should be clearly presented, together with both general and specific limitations, including efforts to mitigate confounding and reporting biases. This would support the reader (researchers and healthcare professionals) into careful interpretation of relevant results. Finally, the declarations should include a statement on data/code availability (including the version of the statistical software used), and if applicable the protocol registration number.

## 5 The READUS-PV guideline: expected benefits and the way forward

We are confident that the adoption of the READUS-PV guideline will provide undisputable advantages for pharmacovigilance stakeholders (Table 1), including researchers, regulators, and reviewers, improving reproducibility and interpretability of DAs (Raschi et al., 2024). Although READUS-PV recommendations should not be considered as a tool to explicitly assess the overall accuracy and validity of published DAs, their adoption can indirectly improve the quality of research by pointing to items that should be addressed during study design, as demonstrated in other fields (Dewey et al., 2019). Ideally, they should be applied to any study employing a disproportionality approach (alone or integrated on more complex study designs) on ICSRs collected within local, regional, national or international databases (of drugs, vaccines, medical devices, food supplements).

**TABLE 1:** Key advantages for pharmacovigilance stakeholders adopting the READUS-PV guideline.

Stakeholder	Advantages
Researcher	Preparation of transparent articles on DA; promote an adequate interpretation of the results and their limitations; support during study conception and design; allow self-assessment and reproducibility by other researchers; plan a pharmaco-epidemiological research based on the results of READUS-PV-compliant DA
Regulator (and clinician)	Better understanding of the results and their limitations, to appreciate the potential regulatory impact and the actual transferability in clinical practice
Reviewer (and Editor)	Increase the speed and accuracy of the peer-review process; facilitate critical appraisal of the results; focus on content (scientific aspects) rather than reporting; support desk rejection vs. external peer-review (this editorial task could be potentially automated, at least partially, through artificial intelligence tools)

We recognize several challenges in achieving widespread, high-quality reporting of DAs (Supplementary Material). The READUS-PV guideline represents the very first step towards this goal. Achieving this goal requires maintaining an ongoing, constructive dialogue among academia, pharmaceutical companies, regulatory agencies, scientific societies, and scientific journals. The endorsement by Pharmacology Journals dealing with pharmacovigilance and publishing a high volume of DAs is particularly important.

The next 3 years are crucial to fully implement the READUS-PV guideline and appreciate its impact on the scientific community and public health. The actual adherence and effectiveness (increased reporting completeness) will be evaluated by dedicated meta-epidemiological research. With time, they will allow a transparent, reproducible pharmacovigilance research, thus reaffirming the role of DA among other irreplaceable post-marketing research tools.

## Data Availability

The original contributions presented in the study are included in the article/Supplementary Material, further inquiries can be directed to the corresponding author.
