# Supplementary material for: The reporting of disproportionality analysis in pharmacovigilance: spotlight on the READUS-PV guideline
Source: Front Pharmacol. 2024 Nov 27;15:1488725. doi: 10.3389/fphar.2024.1488725 (PMC11632231; doi:10.3389/fphar.2024.1488725)
Supplement: Supplementary file 1 [file DataSheet1.PDF]

SWOT analysis

STRENGTHS

It is expected to:

- increase tranparency, completeness, accuracy, interpretation and reproducibility of research through DA

- assist researchers (even without consolidated expertise in pharmacovigilance) during study design

- support the peer-review process

WEAKNESSES

It should not be intended as a guideline on the quality and value (e.g., novelty) of DA

Periodic updates and implementation are expected

The actual adherence and effectiveness (increased reporting completeness) is still to be verified

OPPORTUNITIES

It can be endorsed by a number of clinical and pharmacology Journals, and implemented with feedback from users

It can be adapted/translated at local level, thus supporting regulators and policy makers

It should promote a conscious use and publication of DA, especially in the context of other sources of evidence

THREATS

Endorsement by clinical Journals

Heterogenous implementation by Journals (mandatory checklists vs general statement in the manuscript)

Effective adherence by researchers (perceived fatigue to new reporting requirements)
